# CNV analysis in a large schizophrenia sample implicates deletions at 16p12.1 and *SLC1A1* and duplications at 1p36.33 and *CGNL1*

**DOI:** 10.1093/hmg/ddt540

**Published:** 2013-10-26

**Authors:** Elliott Rees, James T.R. Walters, Kimberly D. Chambert, Colm O'Dushlaine, Jin Szatkiewicz, Alexander L. Richards, Lyudmila Georgieva, Gerwyn Mahoney-Davies, Sophie E. Legge, Jennifer L. Moran, Giulio Genovese, Douglas Levinson, Derek W. Morris, Paul Cormican, Kenneth S. Kendler, Francis A. O'Neill, Brien Riley, Michael Gill, Aiden Corvin, Wellcome Trust Case Control Consortium, Pamela Sklar, Christina Hultman, Carlos Pato, Michele Pato, Patrick F. Sullivan, Pablo V. Gejman, Steven A. McCarroll, Michael C. O'Donovan, Michael J. Owen, George Kirov

**Affiliations:** 1 MRC Centre for Neuropsychiatric Genetics and Genomics, Institute of Psychological Medicine and Clinical Neurosciences, Cardiff University, Cardiff CF24 4HQ, UK,; 2 Stanley Center for Psychiatric Research, The Broad Institute of MIT and Harvard, 7 Cambridge Center, Cambridge, MA 02142, USA,; 3 Department of Genetics, University of North Carolina at Chapel Hill, Chapel Hill, NC, USA,; 4 Department of Psychiatry and Behavioral Sciences, Stanford University, Stanford, CA, USA,; 5 Department of Psychiatry and Neuropsychiatric Genetics Research Group, Institute of Molecular Medicine, Trinity College Dublin, Dublin 2, Ireland,; 6 Department of Psychiatry and Human Genetics, Virginia Institute of Psychiatric and Behavioral Genetics, Virginia Commonwealth University, Richmond, VA, USA,; 7 Department of Psychiatry, Queen's University, BelfastBT71NN, Northern Ireland,; 8 Division of Psychiatric Genomics, Department of Psychiatry, Icahn School of Medicine at Mount Sinai, NY, USA,; 9 Department of Medical Epidemiology and Biostatistics, Karolinska Institutet, Stockholm, Sweden,; 10 Department of Psychiatry and Behavioral Science, Zilkha Neurogenetic Institute, University of Southern California, Los Angeles, CA 90033-0121, USA,; 11 Department of Psychiatry, University of North Carolina at Chapel Hill, Chapel Hill, NC, USA,; 12 Department of Psychiatry and Behavioral Sciences, NorthShore University HealthSystem, Evanston, IL 60201, USA and; 13 Department of Psychiatry and Behavioral Sciences, University of Chicago, Chicago, IL 60637, USA

## Abstract

Large and rare copy number variants (CNVs) at several loci have been shown to increase risk for schizophrenia. Aiming to discover novel susceptibility CNV loci, we analyzed 6882 cases and 11 255 controls genotyped on Illumina arrays, most of which have not been used for this purpose before. We identified genes enriched for rare exonic CNVs among cases, and then attempted to replicate the findings in additional 14 568 cases and 15 274 controls. In a combined analysis of all samples, 12 distinct loci were enriched among cases with nominal levels of significance (*P* < 0.05); however, none would survive correction for multiple testing. These loci include recurrent deletions at 16p12.1, a locus previously associated with neurodevelopmental disorders (*P* = 0.0084 in the discovery sample and *P* = 0.023 in the replication sample). Other plausible candidates include non-recurrent deletions at the glutamate transporter gene SLC1A1, a CNV locus recently suggested to be involved in schizophrenia through linkage analysis, and duplications at 1p36.33 and *CGNL1*. A burden analysis of large (>500 kb), rare CNVs showed a 1.2% excess in cases after excluding known schizophrenia-associated loci, suggesting that additional susceptibility loci exist. However, even larger samples are required for their discovery.

## INTRODUCTION

Copy number variants (CNVs) at several loci are now robustly associated with schizophrenia (1–7). The majority of these are flanked by low copy repeats (LCRs) which mediate their formation through non-allelic homologous recombination (NAHR). Therefore, these CNVs occupy the same genomic locations (i.e. they are recurrent). In addition to these recurrent CNVs, two genes, *NRXN1* and *VIPR2*, have also been associated with schizophrenia when disrupted by non-recurrent CNVs with different breakpoints (8–10). All strongly implicated CNV loci in schizophrenia are rare, CNVs at each locus being found in 0.082–0.59% of cases and even less often in controls (5,6). Due to their rarity, very large sample sizes have been required to identify these loci. For example, the latest CNV locus associated with schizophrenia, a duplication of the Williams–Beuren region, was identified with a sample of over 14 000 cases and 28 000 controls (11). It is logical that further susceptibility loci exist, but have so far escaped identification due to their rarity or lower penetrance.

Most strongly associated schizophrenia CNVs are implicated in other neuropsychiatric disorders, such as autism spectrum disorder (ASD), attention deficit hyperactivity disorder, intellectual disability (ID) and developmental delay (DD) (12–14). There are also many further CNVs that increase susceptibility for ID/DD/ASD (14) that have not been associated with schizophrenia. This could also be due to power limitations: current CNV studies in ID/DD have been much larger than in schizophrenia (5,14) and the rate of these CNVs in schizophrenia is usually lower than in ID/DD (15), making their discovery even harder. Therefore, it is possible that some additional CNVs that are enriched in ID/DD will also increase risk for schizophrenia, if tested in much larger samples.

We reasoned that the best way to discover novel schizophrenia susceptibility loci is by using new, very large schizophrenia samples. We first examined rare CNVs in a discovery sample of 6882 cases and 11 255 controls. These cases and most of the controls have not been used before for the discovery of new loci, except the WTCCC2 subset of the control sample which figures in several papers and most meta-analyses. Loci that showed evidence for association were followed up in the additional 14 568 cases and 15 274 controls, bringing the total number of samples analyzed to ∼48 000.

## RESULTS

### Novel candidate CNV loci

Each gene in the genome was examined for exon-disrupting CNVs in cases and controls. After excluding genes within previously implicated loci (13 loci flanked by LCRs, and two individual genes, *NRXN1* and *VIPR2*, listed in Supplementary Material, Table S3), genes in 37 regions (containing 72 genes) were enriched among our discovery cases with nominal levels of significance (two-sided Fisher's exact *P* < 0.05, Supplementary Material, Tables S5 and S6). We removed four regions from subsequent analyses after manual inspection of their *Z*-scores, Log *R* ratio and B-allele frequency traces found them to be unreliable. The significance for none of the genes would survive a conservative Bonferroni correction for multiple testing of 20 000 genes separately for deletions and duplications (*P* < 1.25 × 10^−6^). Restricting the analysis to only individuals of European ancestry (∼90% of the sample) did not change these results (data not presented). In the replication data, CNVs of the same type (deletions or duplications) at 20 of the 33 loci were more common among cases (Supplementary Material, Table S5) but only one (16p12.1) was nominally significant (without multiple-testing correction). In a combined analysis of the discovery and replication samples, genes in 12 distinct regions remained significant (Cochran–Mantel–Haenszel (CMH) *P* < 0.05, Table 1). Again, the significance for none of these genes would survive genome-wide correction for multiple testing.

**Table 1.** Novel candidate CNV loci

Locus/gene	Location (Mb)	Type	Discovery (case/control)	Replication (case/control)	Discovery *P* (two-sided Fisher)	Replication *P* (one-sided Fisher)	Combined *P* (CMH)	Totals % case/control	Odds ratio (95%CI)
1p36.33 *(GNB1, CALML6, TMEM52, KIAA1751, GABRD)*	chr1:1.85–2.11	Dup	9/0 (0.13%/0%)	5/2 (0.034%/0.013%)	0.00016	0.21	0.0005	0.065%/0.0075%	8.66 (1.97–38.12)
*AQP12A, KIF1A*	chr2:241.63–241.75	Dup	51/39 (0.74%/0.35%)	23/25 (0.16%/0.16%)	0.00042	0.61	0.0028	0.34%/0.24%	1.43 (1.02–2.00)
*ELOVL6*	chr4:110.97–111.12	Dup	4/0 (0.058%/0%)	3/1 (0.021%/0.0065%)	0.021	0.29	0.024	0.033%/0.0038%	8.66 (1.07–70.39)
*FAM149A, FLJ38576, CYP4V2*	chr4:187.05–187.14	Dup	9/0 (0.13%/0%)	1/3 (0.0069%/0.02%)	0.00016	0.93	0.02	0.047%/0.011%	4.12 (1.13–14.99)
*TRIML1, TRIML2*	chr4:189.01–189.07	Del	8/3 (0.12%/0.027%)	4/2 (0.027%/0.013%)	0.026	0.32	0.024	0.056%/0.019%	2.97 (1.05–8.43)
*IRGM, ZNF300, SMIM3*	chr5:150.16–150.30	Del	4/0 (0.058%/0%)	5/1 (0.034%/0.0065%)	0.021	0.099	0.015	0.042%/0.0038%	11.14 (1.41–87.90)
*PHACTR2*	chr6:144–144.15	Dup	5/1 (0.073%/0.0089%)	5/2 (0.034%/0.013%)	0.032	0.21	0.03	0.047%/0.011%	4.12 (1.13–14.99)
*GLIS3*	chr9:3.82–4.3	Del	5/0 (0.073%/0%)	2/0 (0.014%/0%)	0.0079	0.24	0.0084	0.033%/0%	NA (1.06–324.96)
*SLC1A1*	chr9:4.49–4.59	Del	6/1 (0.087%/0.0089%)	4/1 (0.027%/0.0065%)	0.014	0.17	0.0098	0.047%/0.0075%	6.19 (1.36–28.24)
*CGNL1*	chr15:57.67–57.84	Dup	35/25 (0.51%/0.22%)	34/25 (0.23%/0.16%)	0.0019	0.11	0.0019	0.32%/0.19%	1.71 (1.19–2.46)
16p12.1 (7 genes)	chr16:21.95–22.43	Del	13/6 (0.19%/0.053%)	20/9 (0.14%/0.059%)	0.0084	0.023	0.0016	0.15%/0.057%	2.72 (1.48–5.02)
*GALR1*	chr18:74.96–74.98	Dup	5/0 (0.073%/0%)	0/0 (0%/0%)	0.0079	1	0.016	0.023%/0%	NA (0.75–246.1)

Discovery = 6882 cases and 11 255 controls. Replication = 14 568 cases and 15 274 controls. CMH = Cochran–Mantel–Haenszel test. Type: Dup = duplications and Del = deletion. CI = confidence interval. Fisher = Fisher's exact test. The columns for discovery and replication datasets display the numbers of CNVs that intersect exons in the gene (or the gene with the strongest *P*-value in regions that contain multiple genes) and the % of cases and controls affected by these CNVs.

We find the best evidence for the following loci (details in the Supplementary Material):

Deletions at 16p12.1 are the most likely finding as it is the only locus significant in the replication dataset on its own and is a known pathogenic locus associated with ID/DD/ASD (14,16). It includes seven genes disrupted by recurrent deletions flanked by LCRs (Supplementary Material, Fig. S13). In our discovery sample, the deletion was found in 13 cases and 6 controls (0.19 versus 0.053%, two-sided Fisher's exact *P* = 0.0084). In our replication sample, the deletion was found in a further 20 cases and 9 controls (0.14 versus 0.059%, one-sided Fisher's exact *P* = 0.023). A combined analysis of all data found the deletion in 0.15% of cases and 0.057% of controls, CMH *P* = 0.0016, odds ratio (OR) = 2.72, 95% confidence interval (95%CI) = 1.48–5.02 (Table 1).

Our most significant finding involves non-recurrent duplications of five genes at 1p36.33 (*GNB1*, *CALML6*, *TMEM52*, *KIAA1751*, *GABRD*), which partially overlap another known pathogenic locus when deleted: 1p36 (17,18). In the combined data, duplications are found in 0.065% of cases and 0.0075% of controls, CMH *P* = 0.00050, OR = 8.66, 95%CI = 1.97–38.12 (Table 1). As these non-recurrent CNVs have different breakpoints, the five genes have slightly different CNV counts (Supplementary Material, Fig. S3 and Table S5).

The strongest single gene region is *CGNL1* (chr15:57.67–57.84 Mb). In the combined data, *CGNL1* duplications are found in 0.32% of cases and 0.19% of controls, CMH *P* = 0.0019, OR = 1.71, 95%CI = 1.19–2.46 (Table 1 and Supplementary Material, Fig. S12).

Another good single gene candidate is the glutamate transporter *SLC1A1*, where analysis of all data found deletions in 0.047% of cases and 0.0075% of controls, CMH *P* = 0.0098, OR = 6.19, 95%CI = 1.36–28.24 (Table 1 and Supplementary Material, Fig. S11).

### Burden analysis

**Figure 1.**
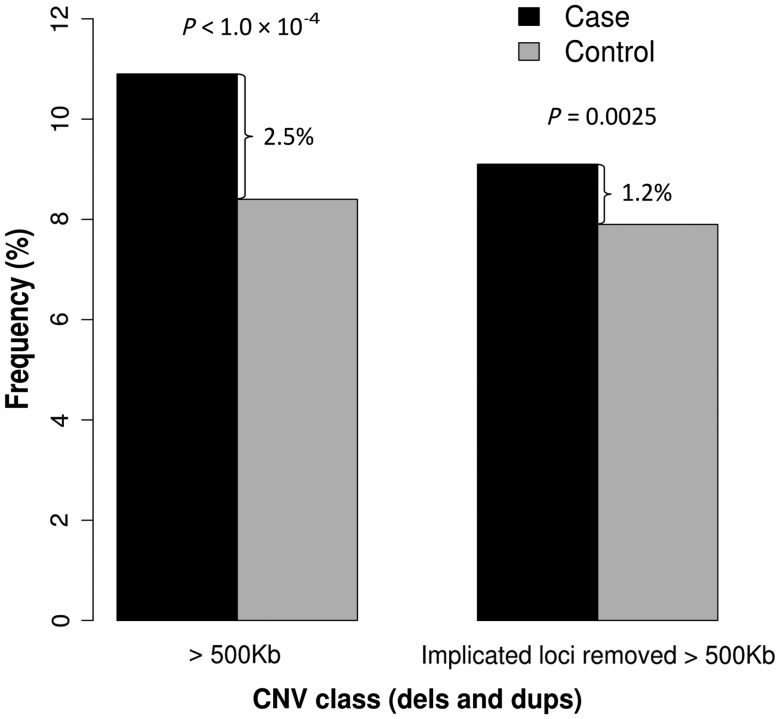
Burden of large (>500 kb) and rare (<1%) CNVs in the discovery sample before and after removing implicated loci.

An increased burden of large, rare CNVs is well established in schizophrenia (2,7). To determine how much of this excess is explained by already implicated loci, we performed a CNV burden analysis in our discovery sample with and without the inclusion of loci implicated in the current study (regions in Table 1) and 15 loci from previous studies (Supplementary Material, Table S3). There is a 2.5% excess of all CNVs >500 kb in cases, of which 1.3% is accounted for by already implicated loci (Fig. 1 and Supplementary Material, Table S3). The remaining 1.2% excess in cases comes mostly from deletions >1 Mb, and from duplications >500 kb (details in Supplementary Material, Table S4 and a full list in Supplementary Material, Table S7).

## Discussion

**Figure 2.**
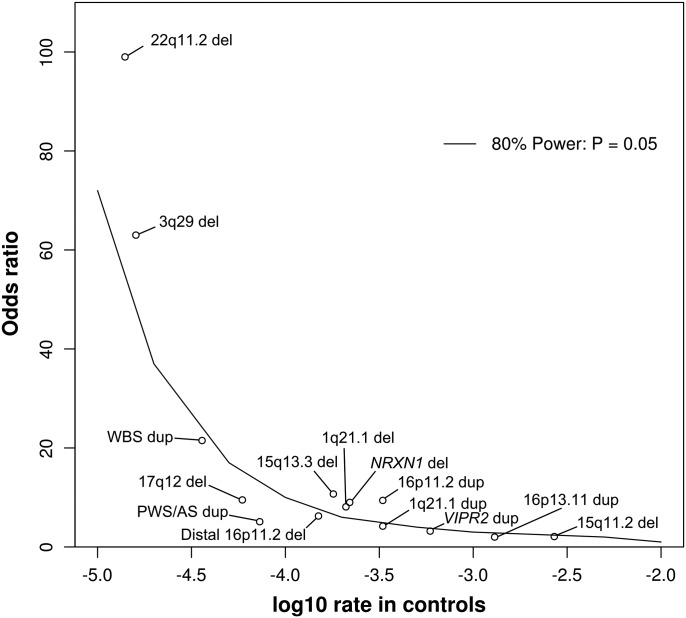
Discovery sample power calculation. The curve represents the point above which our discovery sample had 80% power to detect associations at alpha 0.05. The *x*-axis indicates the frequency of the CNV in controls, on a logarithmic scale (e.g. −4.0 equates to a rate of one CNV per 10 000 people). The *y*-axis is the OR for increasing risk to develop schizophrenia. For reference we indicate the points for previously implicated loci. Frequencies and ORs are from Malhotra and Sebat (5), except for *NRXN1* (4,8); 16p11.2 distal deletion (1) and Williams–Beuren syndrome duplication (11). For the 22q11.2 deletion, there have been no carriers reported among 70 739 controls, leading to a frequency of 0% and OR = infinity. In order to fit more realistic data points into the figure, we added one carrier in controls and scaled down the OR to 99, as no factor can increase the risk for a disease with a population frequency of 1% by more than 100-fold.

It is well established that specific CNVs contribute to schizophrenia susceptibility and moreover that many of the same CNVs also increase risk for other neurodevelopmental disorders (2–4,14,17). Given that large sample sizes are needed to detect rare variants, and even larger samples to provide evidence for association, we performed a CNV association analysis for novel susceptibility loci in the largest schizophrenia CNV dataset reported to date. Our discovery sample is large enough to detect association of typical schizophrenia-associated loci, therefore it appears well placed to serve as a discovery sample for new loci. Supplementary Material, Table S3 shows the frequencies in our discovery sample of 15 CNVs previously implicated from the literature and their strength of association (5,6). Seven of these loci reach a nominal level of significance and would have been retained in the current study for replication had they not already been discovered. Power calculations concur with these results by suggesting that our discovery sample has 80% power with an *α* of 0.05 to detect around half of the previously implicated CNVs (Fig. 2), which were originally discovered with similar sample sizes to ours (2–4,7,19). The significantly increased rate of deletions at *NRXN1* is particularly encouraging, as it is a single gene association, and in the current work we searched for putative risk loci by analyzing our data on a gene-by-gene basis. This indicates that our methods and samples can indeed detect true signals in single genes.

Several studies have shown that patients with schizophrenia have a greater burden of large CNVs compared with controls (2,5). To determine how much of this excess has already been accounted for, we compared the results from a CNV burden analysis before and after removing schizophrenia-associated CNVs. We found that of the 2.5% excess of all CNVs >500 kb in cases, 1.3% comes from loci implicated in the current (0.2%) and previously published studies (1.1%). As our discovery sample has 80% power to detect around half of the previously implicated CNVs (Fig. 2), we speculate that the remaining 1.2% excess yet to be discovered for CNVs >500 kb will be attributed to CNVs with lower population frequencies and/or smaller ORs than those already identified. These will therefore require even larger samples for their discovery, but some are likely to be just singleton observations, where association analysis will fail. We feel that burden analysis of CNVs smaller than 500 kb, even on the high-quality arrays we used, is still subjected to too many potential biases to be reliable (unlike selected individual loci, that can be inspected further, see Materials and Methods and Results), and we are unable to speculate about the precise excess of smaller CNVs. We expect such loci should also exist, but might also have lower ORs and/or frequencies.

Schizophrenia has a high heritability (81% according to the largest review of twin studies) (20). CNVs probably contribute to this heritability; however, they can explain only a small part of it. Our results suggest that only 2.5% of patients (versus 1% of controls) carry a confirmed schizophrenia-associated CNV (Supplementary Material, Table S3), with an additional 1.2% excess burden of large CNVs (Fig. 1). Not all of these would contribute to heritability, as some 16% of the >500 kb CNVs occur *de novo* (21) as do 9–80% of those at specific schizophrenia-associated loci (15,22). In addition, their penetrance is not complete (15), which would lower their contribution to heritability. We cannot speculate on the extent of the contribution of smaller CNVs, but no doubt they contribute as well.

### Deletions at 16p12.1

Although we found no novel genome-wide significant associations, we report deletions at 16p12.1 as the most likely new risk locus. This deletion has previously been observed in a schizophrenia cohort (16), but that study lacked the statistical power to implicate it. Here we show for the first time enrichments with nominal levels of significance in both our discovery and replication cases, resulting in a combined *P*-value of 1.6 × 10^−3^. The deletion has the hallmarks of almost all robustly associated CNVs, in that it is recurrent, is flanked by LCRs, disrupts multiple genes and is a susceptibility locus for developmental disorders (15,16). The clinical features previously associated with 16p12.1 deletions include DD, speech delay, epilepsy and craniofacial and skeletal abnormalities (16). 16p12.1 deletions are found in 0.2% of ID/DD patients (16) and we observe them in 0.15% of schizophrenia cases and 0.057% of controls. The combination of a modest OR (OR = 2.72) for developing schizophrenia and a low frequency could explain why it has not been identified until now (our discovery sample has <80% power to detect its association at *α* 0.05; Fig. 2).

Around a quarter of ID/DD patients with a 16p12.1 deletion also carry an additional pathogenic CNV (defined as a CNV associated with ID/DD or >500 kb) (15,16) and in those that do so, the phenotype is more severe (16). In our discovery sample we found only 1 of 13 cases to also carry an additional pathogenic CNV (a 15q11.2 deletion) and none out of the six controls. Although this lower rate is not statistically significant compared with the ID/DD data, it is plausible that individuals with a second hit are more likely to have ID/DD, while those with only the 16p12.1 deletion are more at risk of developing schizophrenia (23). A structural polymorphism with two configurations is known to affect the orientation of LCRs that mediate the formation of 16p12.1 deletions (23,24). Individuals with the more common configuration have LCRs in direct orientation (23,24), which is a mechanistic requirement for NAHR to form deletions. As this risk configuration is found at different frequencies across European (83%), African (98%) and Asian populations (72%), there is a potential for population stratification to bias 16p12.1 associations (23,24). However, when we restrict our analysis to individuals of European decent, the deletion shows even greater enrichment (13/6307 cases, 5/10 676 controls, two-sided Fisher's exact *P* = 0.0029).

### 1p36.33 duplications

The five genes disrupted by duplications in 0.065% of cases and 0.0075% of controls (CMH *P* = 0.0005) are located within 1.8 Mb of the 10 Mb 1p36 deletion syndrome region, a known pathogenic locus for ID/DD when deleted (17,18). The gene with the strongest *P*-value in this region is *KIAA1751* (*P* = 0.0005), but it is uncharacterized. A more likely candidate is the gamma-aminobutyric acid (GABA) A receptor, delta (*GABRD*) (Supplementary Material, Fig. S3 and Table S5) which has been suggested to be responsible for the neuropsychiatric characteristics seen in 1p36 deletion patients as it is highly expressed in brain and functions as a subunit of GABA-A receptors (25). Another promising candidate is *GNB1*, a member of the previously implicated *N*-methyl-d-aspartic acid (NMDAR) gene pathway (26). It is also possible that the disruption of all five genes increases risk for SCZ.

### *CGNL1* duplication

Our strongest statistical result for CNVs hitting a single gene involves duplications of cingulin-like 1 (*CGNL1*), where we find exons disrupted in 0.32% of cases and 0.19% of controls (CMH *P* = 0.0019). The duplications are mostly identical in size and do not cover the whole gene (Supplementary Material, Fig. S12). *CGNL1* is found at adherent junctions and tight cell–cell junctions and coordinates junction assembly via Rac1 and RhoA GTPases (27,28). In the case–control analysis by Levinson *et al*. (4), duplications of this gene were reported as having suggestive evidence for association with schizophrenia, but this was only presented in their Supplemental Material. That study used the Molecular Genetics of Schizophrenia (MGS) and International Schizophrenia Consortium (ISC) cohorts, similar to our replication sample.

### *SLC1A1* deletions

*SLC1A1* encodes a high-affinity glutamate transporter responsible for inactivating synaptic glutamate and preventing extracellular levels of glutamate from reaching neurotoxic levels (29). Glutamate acts on NMDAR receptors, and a large body of evidence has associated NMDAR dysfunction with schizophrenia (30). We find non-recurrent exonic deletions in 0.047% of cases and 0.0075% of controls (CMH *P* = 0.0098). Recently, Myles-Worsley *et al*. (31) reported a deletion of this gene to co-segregate with schizophrenia and bipolar schizoaffective disorder in a five-generation family, reaching a lod-score of 3.64. To our knowledge, this is the first time a CNV linkage association has received support from a large case–control analysis in schizophrenia. Additional observations of *SLC1A1* deletions in disease cohorts include one exonic deletion in 235 subjects with both SCZ and epilepsy (32), a single exonic deletion in 459 unrelated adults with schizophrenia (33), one exonic deletion among 1637 German patients with schizophrenia or schizoaffective disorder (34) and Cooper *et al*. (17) report an enrichment of *SLC1A1* deletions in neurological, craniofacial and epilepsy cases.

In conclusion, we have used a large sample of patients and controls to discover new CNV susceptibility loci. This sample has the power to detect on its own a large proportion of the previously associated CNV loci (Fig. 2 and Supplementary Material, Table S3). We suggest a role for an additional 12 new loci, but only one of these was significant in our replication sample, and even that result does not withstand correction for multiple testing. Therefore, these results need independent confirmation in further large samples. Excluding loci that are already strongly implicated, an excess burden of large and rare CNVs remains in cases, indicating that there are likely to be further susceptibility genes disrupted by CNVs, but these will be of smaller effect size, or very rare, so would require even larger samples to be identified.

## Materials and Methods

‘The discovery sample’ consisted of 7129 schizophrenic cases (prior to quality control (QC) filtering) from the CLOZUK (*N* = 6558) and the CardiffCOGS (*N* = 571) samples, which have been previously described (6,15,35) but have not yet contributed to any analysis aimed at identifying new CNV loci. Briefly, the CLOZUK sample consists of patients taking the antipsychotic clozapine, a drug reserved in the UK for patients that have not responded to trials of at least two other antipsychotics. To allow for early detection of neutropaenia that can result from treatment with clozapine, patients are required to provide regular blood samples. Through collaboration with Novartis, the manufacturer of a proprietary form of clozapine (Clozaril), we acquired anonymized DNA samples from people with schizophrenia who were taking the drug. Approval by the local ethics committee was granted for the use of these samples in genetic association studies. Patients are aged 18–90, had a recorded diagnosis of treatment resistant schizophrenia, and 71% are male. A higher male ratio is not unusual for samples recruited for genetic studies in schizophrenia: this proportion is 66% in the ISC study (2) and 70% in the MGS study (4). The CardiffCOGS is a sample of clinically diagnosed schizophrenia patients from the UK. Interview with the SCAN instrument (36) and case note review was used to arrive at a best-estimate lifetime diagnosis according to DSM-IV criteria (37). All discovery cases were genotyped at the Broad Institute, Stanley Centre for Psychiatric Research, USA on either Illumina OmniExpress or OmniCombo arrays.

The discovery control cohort consisted of four publicly available, non-psychiatric datasets, totaling 12 080 samples prior to QC (Supplementary Material, Table S2). These datasets were chosen as they were genotyped on Illumina arrays similar to those used for the cases: Illumina Human Omni2.5, Illumina HumanOmni1_Quad or Illumina 1.2M. Further details of these samples are provided in the Supplementary Material.

### CNV calling and QC

Principal component analysis was performed to derive ethnicities of discovery samples. Identity by decent was performed to identify and remove duplicate individuals. For each case and control dataset, Log R Ratios (LRR) and B-allele frequencies were generated using Illumina Genome Studio software (v2011.1) and used to call CNVs with PennCNV (38). CNV calling was performed following the standard protocol and adjusting for GC content. To avoid a cross-platform CNV locus detection bias in the discovery sample, we called CNVs using a consensus set of 520 766 probes that are present on all microarrays used. Samples were excluded if they were found to be an outlier for any one of the following QC metrics: LRR standard deviation, B-allele frequency drift, wave factor and total number of CNVs called per person. The numbers of discovery cases and controls that passed QC are presented in Table 2. All coordinates in this paper are according to UCSC build 37, hg19.

**Table 2.** Number of discovery and replication samples passing QC and their genotyping platforms

Sample	Array	*N* cases	*N* controls
Discovery			
CLOZUK and CardiffCOGs	Illumina OmniExpress/OmniCombo	6882	
Smoking	Illumina Human Omni2.5		1488
Melanoma	Illumina HumanOmni1_Quad		2971
KORA	Illumina Human Omni2.5		1857
WTCCC2	Illumina 1.2M		4939
Total Discovery		6882	11 255
Replication			
MGS EA	Affy 6.0	2215	2556
MGS AA	Affy 6.0	977	881
ISC	Affy 6.0/5.0	3045	3185
BG trios	Affy 6.0	662	662
Irish	Affy 6.0	1377	992
Swedish	Affy 5.0 (3.9%), Affy 6.0 (38.6%), Illumina OmniExpress (57.4%)	4655	6038
African American	Illumina Omni2.5	1637	960
Total replication		14 568	15 274

Discovery control samples were obtained from the following sources: smoking = the genetic architecture of smoking and smoking cessation, dbGaP (phs000404.v1.p1); melanoma = high-density SNP association analysis of melanoma: case–control and outcomes investigation, dbGaP (phs000187.v1.p1); kora = genetic epidemiology of refractive error in the KORA study, dbGaP (phs000303.v1.p1); WTCCC2 = WTCCC2 project samples from National Blood Donors (NBS) Cohort, European Genome-Phenome Archive (EGAD00000000024) and WTCCC2 project samples from the 1958 British Birth Cohort, European Genome-Phenome Archive (EGAD00000000022). MGS = molecular genetics of schizophrenia (EA = European, AA = African)(4), BG trios = Bulgarian trios (26), ISC = International Schizophrenia Consortium (2). Bulgarian probands from the BG trios sample were excluded from the ISC sample, and the Swedish sample does not include individuals who were included in the ISC.

CNVs from samples that passed QC were joined together if the distance separating them was <50% of their combined length using an in-house developed open source program (http://x004.psycm.uwcm.ac.uk/~dobril/combine_CNVs/). CNVs were then excluded if they were either <10 kb, covered by <10 probes, overlapped with LCRs by >50% of their length or had a probe density of <1 SNP/20 kb. CNV loci with a frequency >1% in all samples were excluded using PLINK (39). Finally, all CNVs were validated by the *in silico* median *Z*-score outlier method, described in detail elsewhere (26) and in the Supplementary Material. Briefly, this method uses the median value of all normalized LRR probe intensities within a CNV to assess copy number. All CNVs in regions enriched among cases in our Discovery sample that passed our filtering criteria are available in Supplementary Material, Table S6.

‘Replication samples’ consisted of six independent case/control datasets and one trio dataset, totaling of 14 568 cases and 15 274 controls after QC: MGS (4), split for samples with a European American or African American ancestry; International Schizophrenia Consortium (2); Bulgarian trios (BG trios) (26); Irish (40); Swedish (41); and African American from the Genomic Psychiatry Cohort in the United States (42). Bulgarian probands from the BG trios sample were excluded from the ISC sample, and the Swedish sample does not include individuals who were part of the ISC. The number of replication samples that passed QC and the arrays they were genotyped on are presented in Table 2. Further details on genotyping and QC of these datasets are presented in the Supplementary Material.

### Statistical analysis

To identify novel risk loci we adopted a gene-based approach. Each gene in the genome was examined for exon-disrupting CNVs using refseq gene coordinates (downloaded from the UCSC genome browser, includes non-coding RNAs). Deletions and duplications were counted and analyzed separately. Genes that reached nominal levels of significance in the discovery sample with a two-sided Fisher's exact test (*P* < 0.05) were further analyzed with a one-sided Fisher's exact test in the replication data. The combined sample of all available data was analyzed with a CMH test, stratified by dataset: discovery sample as a single dataset and each of the seven replication datasets as separate samples, as shown in Table 2 (the MGS sample was split for ethnicity). This gene-wise approach can capture signal from both single gene enrichments, such as *NRXN1* deletions, and larger recurrent events through contiguous gene enrichments.

The burden of large and rare CNVs in cases versus controls was evaluated with a one-sided test and 10 000 permutations using PLINK (39). The analysis was stratified by CNV size (>500, 500–1 and >1 Mb) and CNV type (all CNVs, deletions only and duplications only). All CNVs >500 kb used in the burden analysis are listed in Supplementary Material, Table S7.

In order to determine the effect size and population frequency of CNVs that our discovery sample had 80% power to detect with an *α* of 0.05 (Fig. 2), we used an online open source genetic power calculator (http://pngu.mgh.harvard.edu/~purcell/gpc/) (43).

## SUPPLEMENTARY MATERIAL

Supplementary Material is available at *HMG* online.

## FUNDING

The work at Cardiff University was funded by Medical Research Council (MRC) Centre (G0800509) and Program Grants (G0801418), the European Community's Seventh Framework Programme (HEALTH-F2-2010-241909 (Project EU-GEI), an MRC PhD Studentship to E.R., a Clinical Research Fellowship to J.T.R.W. from the MRC/Welsh Assembly Government and the Margaret Temple Award from the British Medical Association. The 7129 SCZ samples from the ‘discovery sample’ were genotyped at the Broad Institute, USA, funded by a philanthropic gift to the Stanley Center for Psychiatric Research. Funding support for the Swedish study was provided by NIMH R01 MH077139 (P.S.), NIMH R01 MH095034 (P.S.), the Stanley Center for Psychiatric Research, the Karolinska Institutet, Karolinska University Hospital, the Swedish Research Council, an ALF grant from Swedish County Council, the Söderström Königska Foundation and the Netherlands Scientific Organization (NWO 645-000-003). Funding to pay the Open Access publication charges for this article was provided by the MRC UK and the Wellcome Trust.

## Supplementary Material

Supplementary Data
