# Supplementary material for: CNV analysis in a large schizophrenia sample implicates deletions at 16p12.1 and SLC1A1 and duplications at 1p36.33 and CGNL1
Source: Hum Mol Genet. 2013 Oct 26;23(6):1669–76. doi: 10.1093/hmg/ddt540 (PMC3929090; doi:10.1093/hmg/ddt540)
Supplement: Supplementary Data [file supp_23_6_1669__index.html]

CNV analysis in a large schizophrenia sample implicates deletions at 16p12.1 and SLC1A1 and duplications at 1p36.33 and CGNL1 — Supplementary Data 

# CNV analysis in a large schizophrenia sample implicates deletions at 16p12.1 and *SLC1A1* and duplications at 1p36.33 and *CGNL1*

## Supplementary Data

Supplementary Data

**Files in this Data Supplement:**

- Supplementary Data - Pdf file
- Supplementary Table 5 - xlsx file
- Supplementary Table 6 - xlsx file
- Supplementary Table 7 - xlsx file
