# Supplementary material for: CNV analysis in a large schizophrenia sample implicates deletions at 16p12.1 and SLC1A1 and duplications at 1p36.33 and CGNL1
Source: Hum Mol Genet. 2013 Oct 26;23(6):1669–76. doi: 10.1093/hmg/ddt540 (PMC3929090; doi:10.1093/hmg/ddt540)
Supplement: Supplementary Data [file supp_ddt540_ddt540supp.pdf]

Supplementary Material

CNVs analysis in a large schizophrenia sample implicates deletions at 16p12.1 and *SLC1A1* and duplications at 1p36.33 and *CGNL1*

1. Sample description
2. Discovery sample quality control
3. Previously implicated CNV regions
4. CNV burden analysis
5. UCSC tracks of significant loci
6. Acknowledgments
7. References

1. Sample Description

Discovery sample: The 7129 discovery cases came from samples we call the CLOZUK (n=6,558) and CardiffCOGS (n=571) series which have in part been described previously (1-3). The CLOZUK sample consists of patients taking clozapine, who provide regular blood samples to allow early detection of adverse effects of that treatment. Through collaboration with Novartis, the manufacturer of a proprietary form of clozapine (Clozaril), we acquired blood from people with schizophrenia who were taking the drug via the central processing labs of a clozapine blood monitoring service. After the samples had been used to complete the necessary laboratory tests, unused fractions were sent to Tepnel Life Sciences (Paisley, UK) for DNA extraction. Samples were anonymous, only basic demographic and diagnostic details being made available. Subjects (71% male) were UK residents, aged 18-90 with a recorded diagnosis of treatment resistant schizophrenia according to the clozapine registration forms completed by the treating psychiatrists. In the UK, treatment resistant schizophrenia implies a lack of satisfactory clinical improvement to adequate trials of at least two other antipsychotics. Approval by the local ethics committee was granted for the use of these samples in genetic association studies.

The CardiffCOGS is a sample of clinically diagnosed schizophrenic patients from the UK. Interview with the SCAN instrument (4) and case note review was used to arrive at a best-estimate lifetime diagnosis according to DSM-IV criteria (5).

All cases were genotyped on either HumanOmniExpress-12v1 or HumanOmniExpressExome-8v1 arrays at the Broad Institute, Cambridge, Massachusetts.

All controls for the discovery sample were downloaded with the relevant approvals for our study from the online repositories Database of Genotypes and Phenotypes (dbGaP) and the

European Genome-Phenome Archive (EGA). The four non-psychiatric control datasets obtained, totalling 12,080 samples, are summarised in Table S1. We purposefully selected datasets that were genotyped on high density Illumina arrays to maximise probe overlap with the Illumina arrays used to genotype cases.

**Dataset**	**Source (accession ID)**	**Array (N probes)**	**N Samples**
Schizophrenia Batch 1	Broad Institute	HumanOmniExpress-12v1 (730,525)	2,469
Schizophrenia Batch 2	Broad Institute	HumanOmniExpressExome-8v1 (951,117)	3,621
Schizophrenia Batch 3	Broad Institute	HumanOmniExpressExome-8v1 (951,117)	1,039
The Genetic Architecture of **Smoking** and Smoking Cessation	dbGaP (phs000404.v1.p1)	Illumina HumanOmni2.5 (2,443,179)	1,491
High Density SNP Association Analysis of **Melanoma**: Case-Control and Outcomes Investigation	dbGaP (phs000187.v1.p1)	Illumina HumanOmni1_Quad_v1-0-B (1,051,295)	3,102
Genetic Epidemiology of Refractive Error in the **KORA** Study	dbGaP (phs000303.v1.p1)	Illumina HumanOmni2.5 (2,443,179)	1,869
**WTCCC2** project samples from National Blood Donors (NBS) Cohort	EGA (EGAD00000000024)	Illumina 1.2M (1,238,733)	2,697

**WTCCC2** project samples from 1958 British Birth Cohort	EGA (EGAD00000000022)	Illumina 1.2M (1,238,733)	2,921

Table S1. Summary of discovery cases and controls. Number of samples are those before quality control filtering.

Principal component analysis (PCA) was performed to derive the ancestries of the discovery cases and controls by combining the data with Hapmap genotypes. Samples were stratified into those from a European (6,530 cases, 11,434 controls), African (263 cases, 478 controls) or ‘other’ (336 cases, 108 controls) origin.

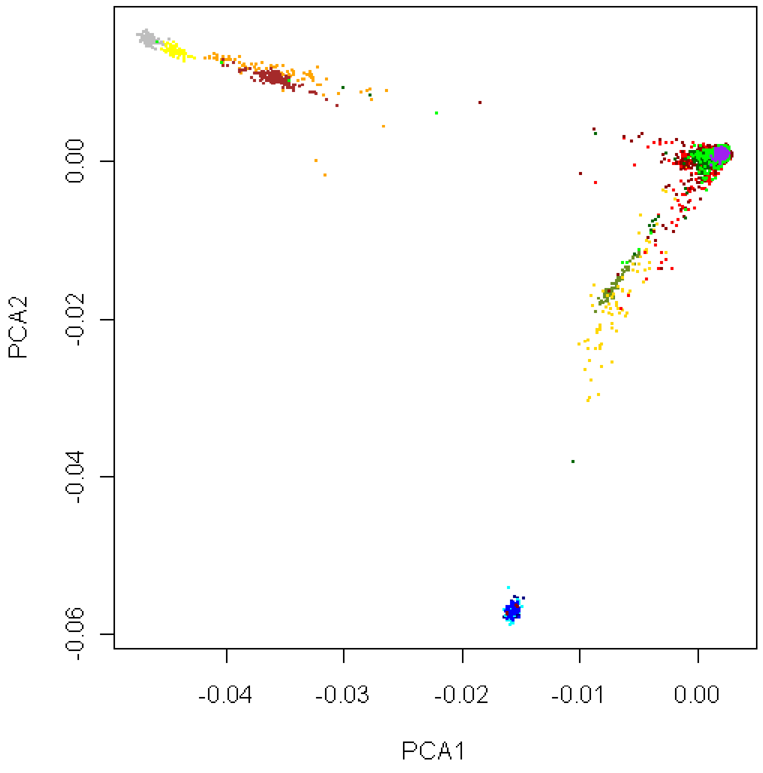

Figure S1. PCA plots for PCA1 and PCA2 for the samples in the discovery set, together with HapMap individuals. Different samples and self-reported ethnic origins from different samples are shown in different colours. At the bottom are individuals from China (light blue)

and Japan (dark blue). On the top left are individuals of African origin, with grey being Yoruba, Nigeria and yellow being Lihua, Kenya. Most individuals, who are white Europeans, are at the top right-hand corner (with PCA1 and PCA2 values of ~0.00). Individuals next to them are of mixed or Indian origin, and they were classed as “others” in our analysis.

Replication samples

Molecular Genetics of Schizophrenia (MGS): Details of the MGS cohort have been described elsewhere (6). We processed the raw data and interrogated CNVs in 2,215 cases and 2,556 controls of European American ancestry and 977 cases and 881 controls of African American ancestry that passed our quality control. All schizophrenic patients met DSM-IV criteria(5) for schizophrenia or schizoaffective disorder. The samples were genotyped at the Broad Institute, Cambridge, Massachusetts, using Affymetrix 6.0 genotyping arrays. CNVs were called using the Birdsuite algorithm (7).

International Schizophrenia Consortium (ISC): Details of the ISC sample have been previously published (8). The sample consists of six European populations genotyped at the Broad Institute, Cambridge, Massachusetts, using Affymetrix 6.0 or Affymetrix 5.0 genotyping arrays. We analysed CNVs in 3,045 cases and 3,185 controls. The Bulgarian cases that are also part of the Bulgarian trio sample (below) are excluded from the analysis of the ISC samples.

Irish sample: Details of these samples have been published previously (9). WTCCC2 samples that overlapped with our discovery sample were excluded. Calls in the Irish schizophrenia sample were created using Birdseye from Birdsuite (version 1.5.5)(7) for autosomes and we excluded calls where lengths were <100kb or >10Mb, or LOD score <10. We excluded CNVs with at least 50% overlap with other regional CNVs present in 1% or

more of the samples. We excluded individuals with >30 CNV calls, or a total CNV length >10Mbp. Calls from plates containing fewer than 40 samples were also excluded.

Swedish sample: Subjects. See Ripke et al. for full description(10). Briefly, all procedures were approved by ethical committees in Sweden and in the US, and all subjects provided written informed consent (or legal guardian consent and subject assent). Cases with schizophrenia were identified via the Swedish Hospital Discharge Register (11, 12) which captures all public and private inpatient hospitalizations. The register is complete from 1987 and augmented by psychiatric data from 1973-86. The register contains ICD discharge diagnoses (13-15) made by attending physicians for each hospitalization (16-19). Case inclusion criteria included ≥2 hospitalizations with a discharge diagnosis of schizophrenia, both parents born in Scandinavia, and age ≥18 years. Case exclusion criteria were hospital register diagnosis of any medical or psychiatric disorder mitigating a confident diagnosis of schizophrenia as determined by expert review, and included removal of 3.4% of eligible cases due to the primacy of another psychiatric disorder (0.9%) or a general medical condition (0.3%), or uncertainties in the Hospital Discharge Register (e.g., contiguous admissions with brief total duration, 2.2%). The validity of this case definition of schizophrenia is strongly supported. Controls were selected at random from Swedish population registers with the goal of obtaining an appropriate control group and avoiding "super-normal" controls (20). Control inclusion criteria were: never hospitalized for schizophrenia or bipolar disorder (given evidence of genetic overlap with schizophrenia), (21-23) both parents born in Scandinavia, and age ≥18 years. The sample was approximately representative of the Swedish populace in regard to county of birth.
Genotyping, quality control, and imputation. DNA was extracted from peripheral blood samples at the Karolinska Institutet Biobank. Samples were genotyped in six batches at the Broad Institute using Affymetrix 5.0 (3.9%), Affymetrix 6.0 (38.6%), and Illumina OmniExpress (57.4%) chips according to the manufacturers' protocols. Genotype calling, quality control, and imputation were done in four sets corresponding to data from Affymetrix

5.0 (Sw1), Affymetrix 6.0 (Sw2-4), and the OmniExpress batches (Sw5, Sw6). Genotypes were called using Birdsuite (Affymetrix) or BeadStudio (Illumina). The quality control parameters applied were: SNP missingness < 0.05 (before sample removal); subject missingness < 0.02; autosomal heterozygosity deviation; SNP missingness < 0.02 (after sample removal); difference in SNP missingness between cases and controls < 0.02; and deviation from Hardy-Weinberg equilibrium ($P < 10^{-6}$ in controls or $P < 10^{-10}$ in cases).

The Birdseye tool in Birdsuite(7) was applied to intensity data from SNP and CNV probes. The Birdseye algorithm uses a hidden Markov model (HMM) approach to find regions of variable copy number in a sample. Model priors were generated for each genotyping platform. All genomic positions were mapped to the hg19 coordinates.

A multi-step quality control (QC) procedure was implemented in order to assemble a high-quality rare CNV callset. Samples were excluded if they failed SNP QC or if they had > 40 CNV calls or > 10Mb of CNVs(8). CNVs were excluded if they were of low confidence (LOD <10, size < 20kb, or spanning < 10 probes) or if they overlapped large genomic gaps (≥1kb overlap). Any CNVs that appeared to be artificially split by the HMM were annealed. Next, we imposed a 1% frequency threshold by removing any CNV with > 50% of its length spanning a region with CNVs from >1% of total samples as implemented in PLINK(24). After excluding samples which overlapped with the ISC dataset, CNVs were interrogated in 4,655 cases and 6,038 controls.

African American sample: The Genomic Psychiatry Cohort (GPC) is a clinical cohort of patients enrolled at sites across the United States, in a collaboration directed by Drs. Michele and Carlos Pato at USC. Psychiatric diagnoses were made through personal interviews and review of the medical records. Interviews were performed by trained clinicians using a structured psychiatric interview instrument, the Diagnostic Interview for Psychosis and Affective Disorder (DI-PAD), to assess participants. The DI-PAD is based on the Diagnostic Interview for Genetic Studies (DIGS) and includes 90 phenomenological symptom items that are used to arrive at final diagnoses under various diagnostic criteria.

Clinicians reviewed diagnoses that were based on DSM-IV (5). Cases were included in the current study if they met criteria for schizophrenia or schizoaffective disorder. Individuals without a personal or family history of psychosis or mania were eligible to participate as controls. In the current study, we genotyped samples from the GPC cohort members with self-reported African American ancestry. Controls were asked to complete a questionnaire to assess their psychiatric history and the psychiatric history of all first-degree relatives. Individuals reporting any lifetime symptoms indicative of psychosis or mania were excluded as control participants

CNVs were called on all samples using PennCNV and NCBI37/hg19 coordinates. The following samples were removed: duplicate individuals, first degree relatives (if discordant phenotypes, always the control was removed), individuals with more than 2% missing genotypes, individuals with more than 60% European ancestry, individuals with more than 10Mbp of the genome estimated as CNV. After quality control, CNVs were analysed in 1,637 cases and 960 controls.

2. Discovery sample quality control

Raw intensity data from each case/control dataset were independently processed and analysed to account for potential batch effects. Log R ratios (LRR) and B-allele frequencies were generated using Illumina Genome Studio software (v2011.1). CNVs were called using the PennCNV calling algorithm, following the standard protocol and adjusting for GC content. CNVs were called using the 520,766 probes common to all discovery arrays to avoid a cross-platform CNV locus detection bias. Samples were excluded if for any one of the following QC metrics they represented an outlier in their source dataset: LRR standard deviation, B-allele frequency drift, wave factor and total number of CNVs called per person. Table S2 shows the number of samples that passed and failed QC from each discovery dataset. As some of the data had been already filtered for quality before they were downloaded, the proportions of failed samples across the datasets are not comparable.

			Ethnicity		
**Sample**	**Total Excluded**	**Total Retained**	**European (retained)**	**African (retained)**	**Other (retained)**
SCZ	247	6882	6530 (6307)	263 (251)	336 (324)
Smoking	3	1488	939 (938)	478 (478)	74 (72)
Melanoma	131	2971	3086 (2955)	0	16 (16)
NBS_WTCCC1	140	1165	1297 (1159)	0	8 (6)
58_WTCCC1	152	1248	1398 (1247)	0	2 (1)
NBS_WTCCC2	182	1210	1386 (1204)	0	6 (6)
58_WTCCC2	205	1316	1519 (1315)	0	2 (1)
KORA	12	1857	1869 (1857)	0	0
Total	1072	18,137	18,024 (16,982)	741 (729)	444 (426)

Table S2. Number of case and control discovery samples, and their ethnicities before and after QC.

Following the exclusion of poorly performing samples, we performed quality control on the called CNVs. Firstly, CNVs in the same individual were joined if the distance separating them was less than 50% of their combined length using a custom developed open source programme (http://x004.psycm.uwcm.ac.uk/~dobril/combine_CNVs/). All CNVs were then excluded if they were covered by less than 10 probes, were less than 10kb in length, overlapped with low copy repeats by more than 50% of their length, or had a probe density (calculated by dividing the size of the CNV by the number of probes covering it) greater than 1 probe/20kb. CNV loci with a frequency > 1% of the total discovery sample were excluded using PLINK (24).

The remaining rare CNVs were required to pass a **median Z-score outlier method** of validation. This method is detailed in Kirov et al, 2012 (25) and Rees et al (3). Briefly, each probe intensity within an individual is converted to a Z-score, which is the probe intensity standardised across all probes within that individual, and then standardised for that probe across all individuals. These rounds of standardisation help reduce noise created by natural fluctuations in probe intensity. A median Z-score value for all probes within a putative CNV region is used to assess copy number, with true deletions and duplications represented as outliers in the samples median Z-score distribution. Each CNV in every individual was assigned a Z-score. CNVs with Z-scores of <-6 were accepted as true deletions, while those with Z-sores of >+3 were accepted as duplications. The Z-score histograms of CNVs with marginal Z-Scores (deletion Z-score between -4 and -6 and duplication Z-score between +2 and +3) were manually inspected, and from these CNVs the LRR and B-allele frequencies of those with ambiguous Z-scores were visually inspected with the Illumina GenomeStudio v2011.1 software. This resulted in 2,569 CNVs being filtered out from the data.

3. Previously implicated CNV regions.

To identify novel CNV associations, we excluded from our analysis regions previously implicated in schizophrenia. These regions, along with the number of CNVs observed in our discovery sample, are presented in Table S3. Some or all of the 4,939 WTCCC2 control samples have been included in previous reviews, or in papers that implicated these loci, so the numbers for the control populations are not entirely independent from previous reports. The evidence that these loci are implicated in SZ is presented in Rees et al (3). Table S3 is based on the data presented in that publication, but not identical to it, as here we include the WTCCC2 controls, that were not used in Rees et al (3). We present the full data here only in order to demonstrate the power of the current sample to detect real associations.

Locus	Position (Mb)	N (frequency) of CNVs in 6,882 cases	N (frequency) of CNVs in 11,255 controls	p-value (Fisher Exact test, 2-sided)
1q21.1 del	chr1:146,57-147,39	12 (0.17%)	4 (0.036%)	0.0034
1q21.1 dup	chr1:146,57-147,39	8 (0.12%)	7 (0.062%)	0.29
NRXN1 exonic del	chr2:50,15-51,26	11 (0.16%)	3 (0.027%)	0.0036
3q29 del	chr3:195,73-197,34	4 (0.058%)	0 (0%)	0.021
WBS dup	chr7:72,74-74,14	3 (0.044%)	1 (0.0089%)	0.16
VIPR2 dup	chr7:158,82-158,94	1 (0.015%)	11 (0.098%)	0.037*
15q11.2 del	chr15:22,80-23,09	44 (0.64%)	47 (0.42%)	0.051
Prader-Willi/ Angelman dup	chr15:24,82-28,43	8 (0.12%)	0 (0%)	0.00043
15q13.3 del	chr15:31,13-32,48	4 (0.058%)	3 (0.027%)	0.44
16p13.11 dup	chr16:15,51-16,30	24 (0.35%)	21 (0.19%)	0.044
16p11.2 distal del	chr16:28,82-29,05	0 (0%)	4 (0.036%)	0.30*

16p11.2 dup	chr16:29,64-30,20	27 (0.39%)	2 (0.018%)	6.9×10^{-10}
17p12 del	chr17:14,16-15,43	4 (0.058%)	5 (0.044%)	0.74
17q12 del	chr17:34,81-36,20	1 (0.015%)	0 (0%)	0.38
22q11.2 del	chr22:19,02-20,26	20 (0.29%)	0 (0%)	3.8×10^{-9}
Total		171 (2.49%)	108 (0.96%)	2.4×10^{-15}

Table S3: CNVs in loci previously implicated in SZ.

* indicates a locus where the trend is for a higher rate of the CNV in controls.

4. CNV burden analysis

Table S4 shows the results of a 1-sided CNV burden analysis with 10,000 permutations (100,000 for loci with p<0.001), implemented with PLINK(24), for all CNVs in the discovery samples, as well as for CNVs with previously implicated loci removed. All CNVs > 500Kb that were included in the burden analysis are provided as an external dataset in table S7.

CNV Type	Size range	All CNVs			Implicated loci removed		
		Case Freq (N CNV)	Con Freq (N CNV)	% Excess (P value)	Case Freq (N CNV)	Con Freq (N CNV)	% Excess (P value)
Del + Dup	>500kb	10.9% (751)	8.4% (940)	2.5% (<0.0001)	9.1% (627)	7.9% (890)	1.2% (0.0025)
Del	>500kb	2.8% (194)	1.8% (205)	1% (<0.0001)	2.0% (141)	1.7% (188)	0.3% (0.039)
Dup	>500kb	8.1% (557)	6.5% (735)	1.6% (0.0004)	7.1% (486)	6.2% (702)	0.9% (0.017)
Del + Dup	500kb – 1Mb	6.8% (467)	5.8% (650)	1% (0.0033)	6.2% (429)	5.7% (638)	0.5% (0.068)
Del	500kb – 1Mb	1.3% (90)	1.2% (135)	0.1% (0.29)	1.2% (83)	1.2% (130)	0% (0.41)
Dup	500kb – 1Mb	5.5% (377)	4.6% (515)	0.9% (0.0054)	5.0% (346)	4.5% (508)	0.5% (0.064)
Del + Dup	> 1Mb	4.1% (284)	2.6% (290)	1.5% (<0.0001)	2.9% (198)	2.2% (252)	0.7% (0.0044)
Del	> 1Mb	1.5% (104)	0.6% (70)	0.9% (<0.0001)	0.8% (58)	0.5% (58)	0.3% (0.0058)
Dup	> 1Mb	2.6% (180)	2.0% (220)	0.6% (0.0027)	2.0% (140)	1.7% (194)	0.3% (0.077)

Table S4. Burden of large CNVs before and after removing implicated loci. CNVs are stratified by type (all, deletions only, duplications only) and size (500kb – 1Mb and > 1Mb).

5. UCSC tracks of significant loci

Location of CNVs that remained significant with a combined discovery/replication Cochran-Mantel-Haenszel test (Table 1 in the main text). The CNV counts in all datasets for the full 62 genes that reached nominal levels of significance in the discovery sample are provided as an external dataset in table S5.

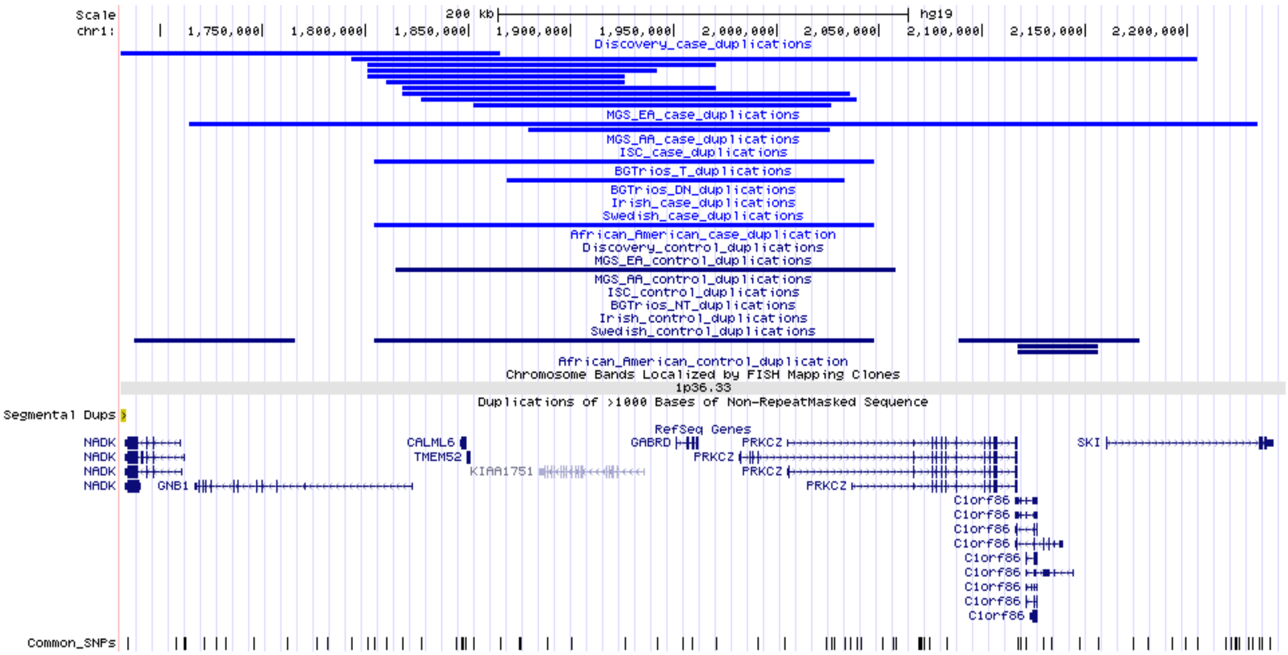

Figure S2. Duplications at 1p36.33

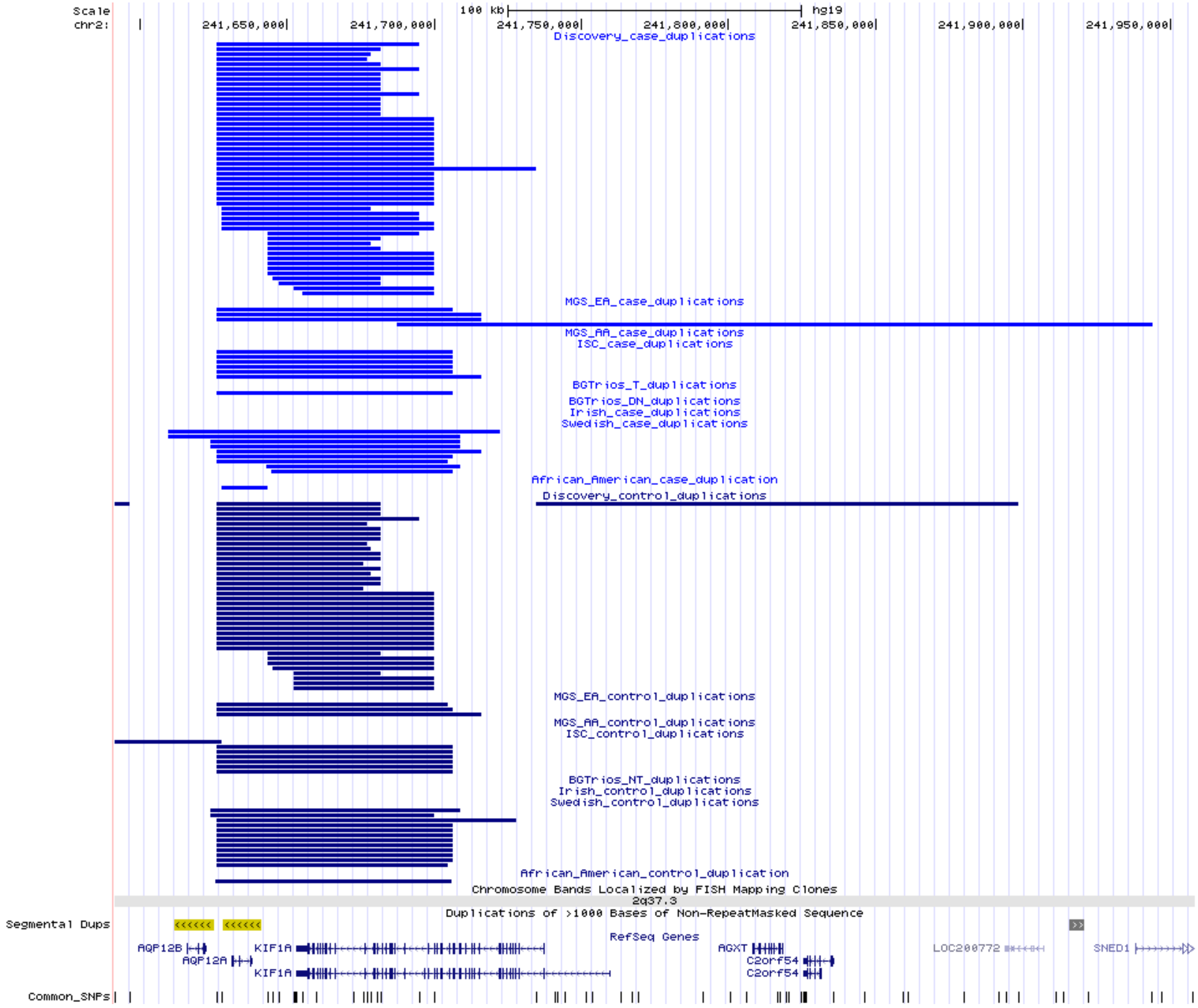

Figure S3. Duplications of *AQP12A* and *KIF1A.*

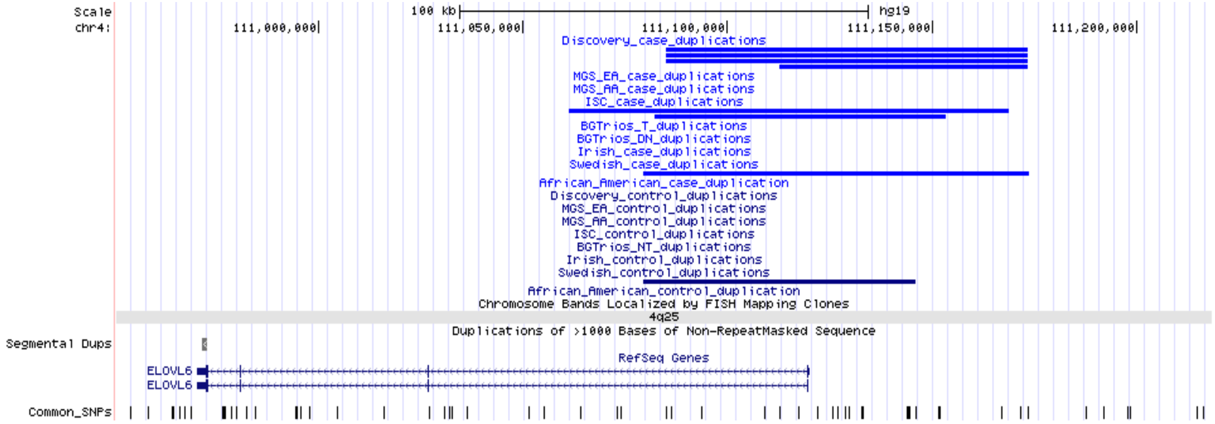

Figure S4. Duplications of *ELOVL6.*

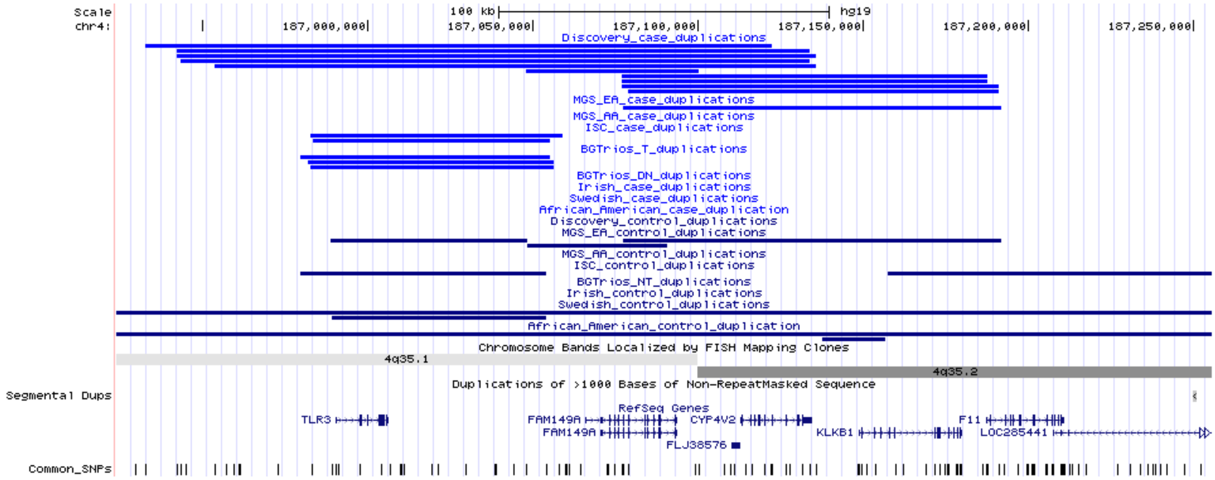

Figure S5. Duplications of *FAM149A*, *FLJ38576* and *CYP4V2*

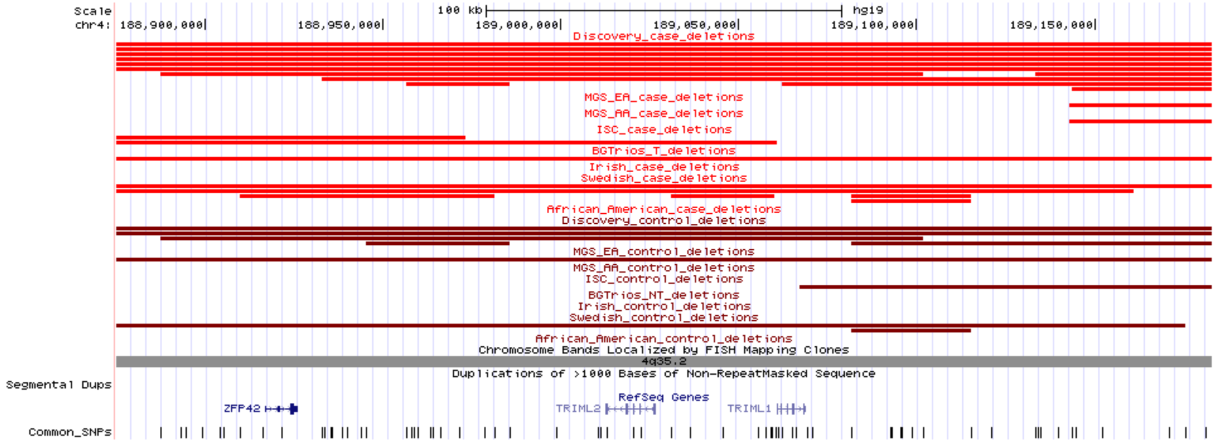

Figure S6. Deletions of *TRIML1* and *TRIML2*

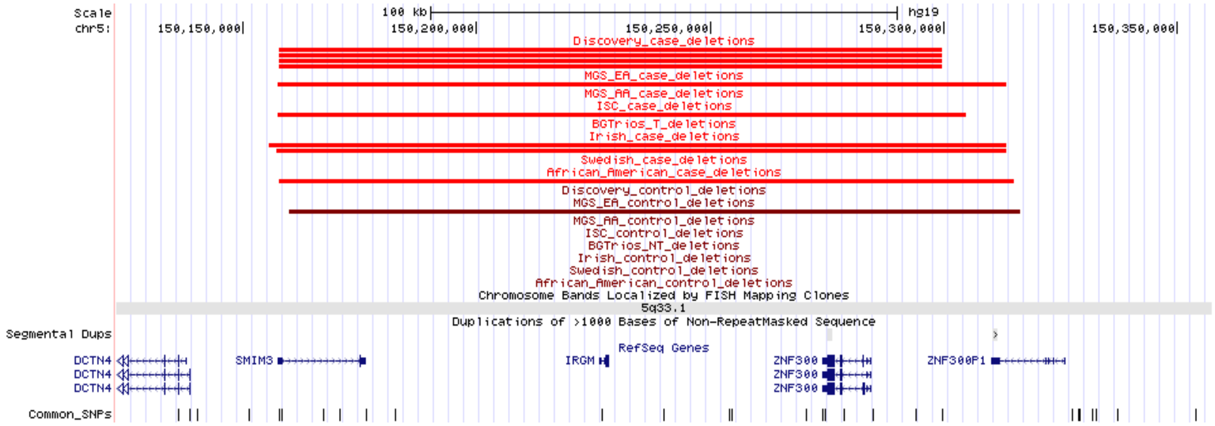

Figure S7. Deletions of *IRGM, ZNF300* and *SMIM3.*

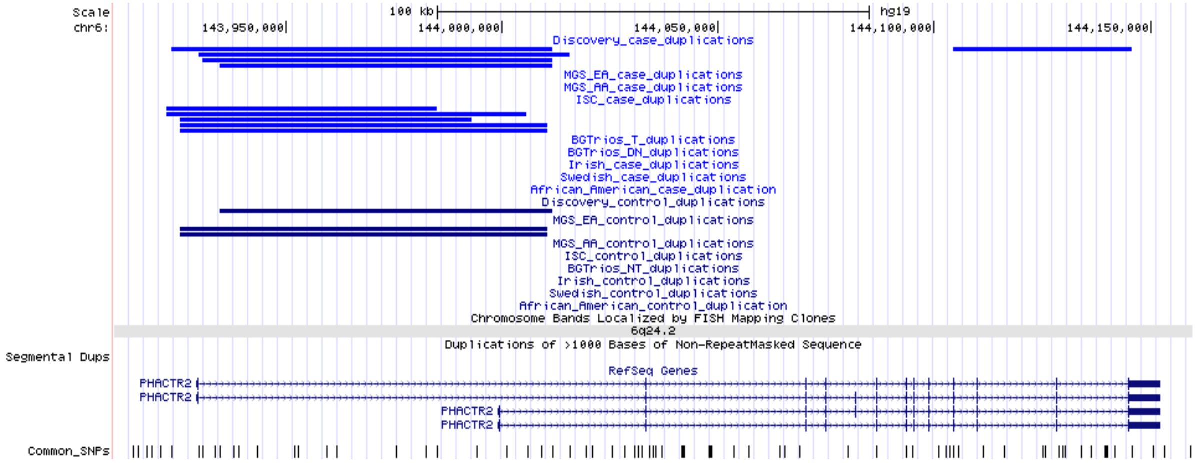

Figure S8. Duplications of *PHACTR2.*

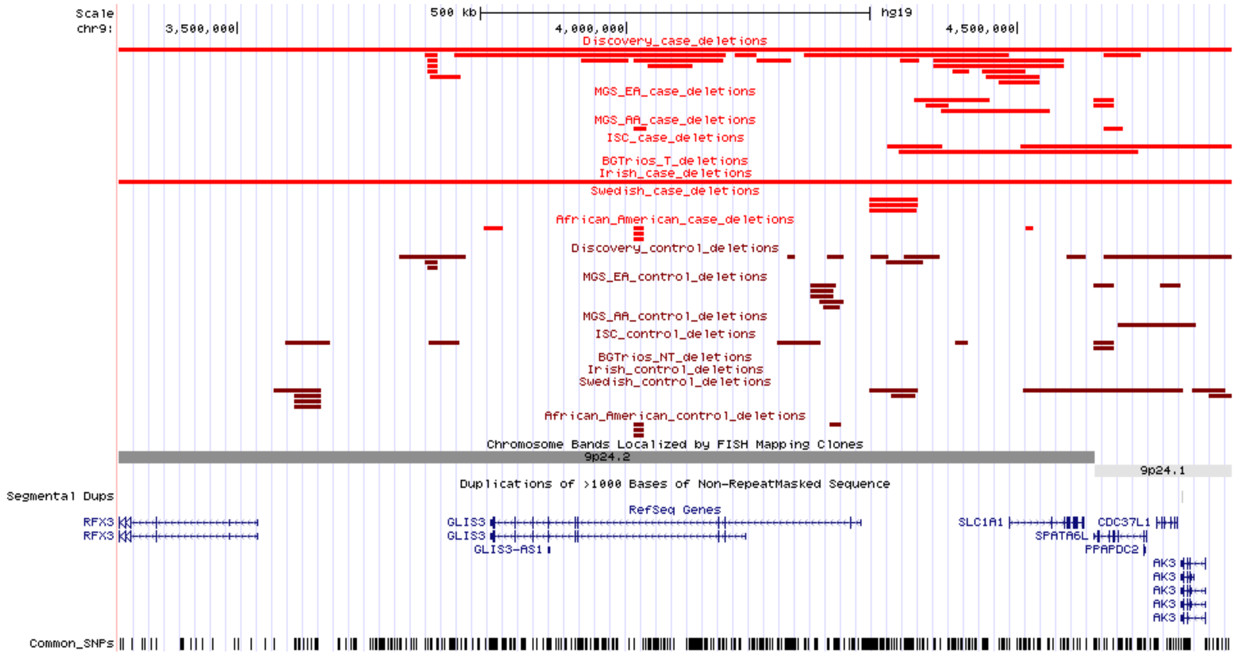

Figure S9. Deletions of *GLIS3*.

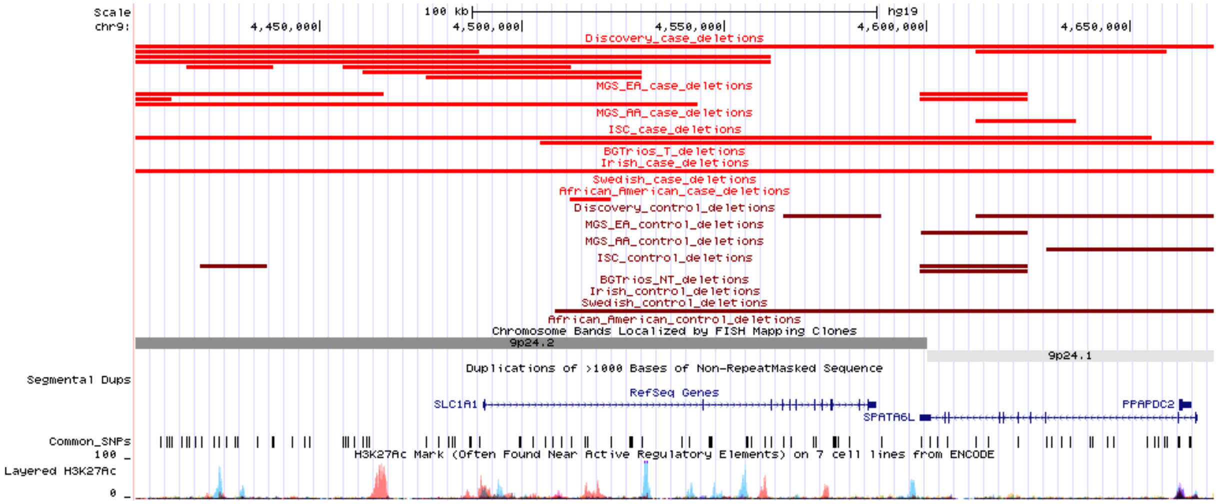

Figure S10. Deletions of *SLC1A1*.

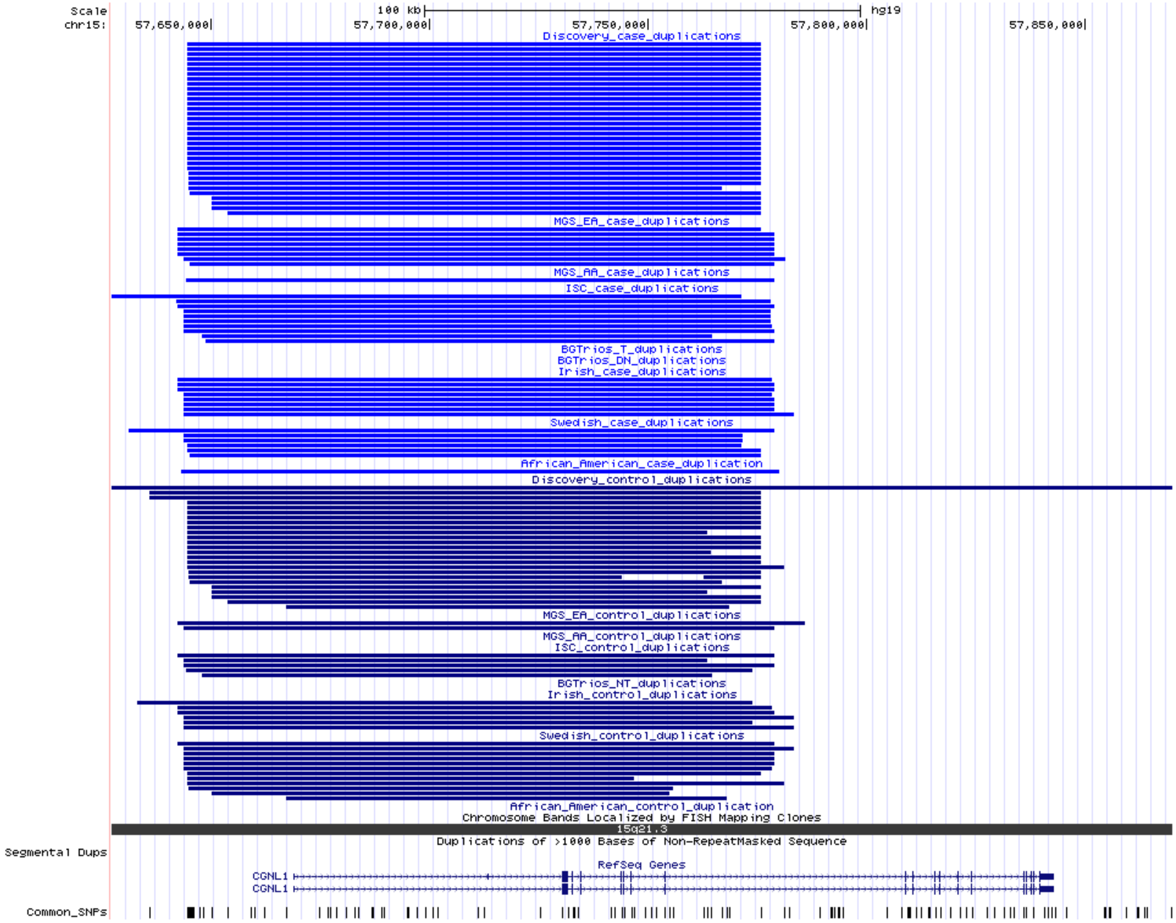

Figure S11. Duplications of *CGNL1.*

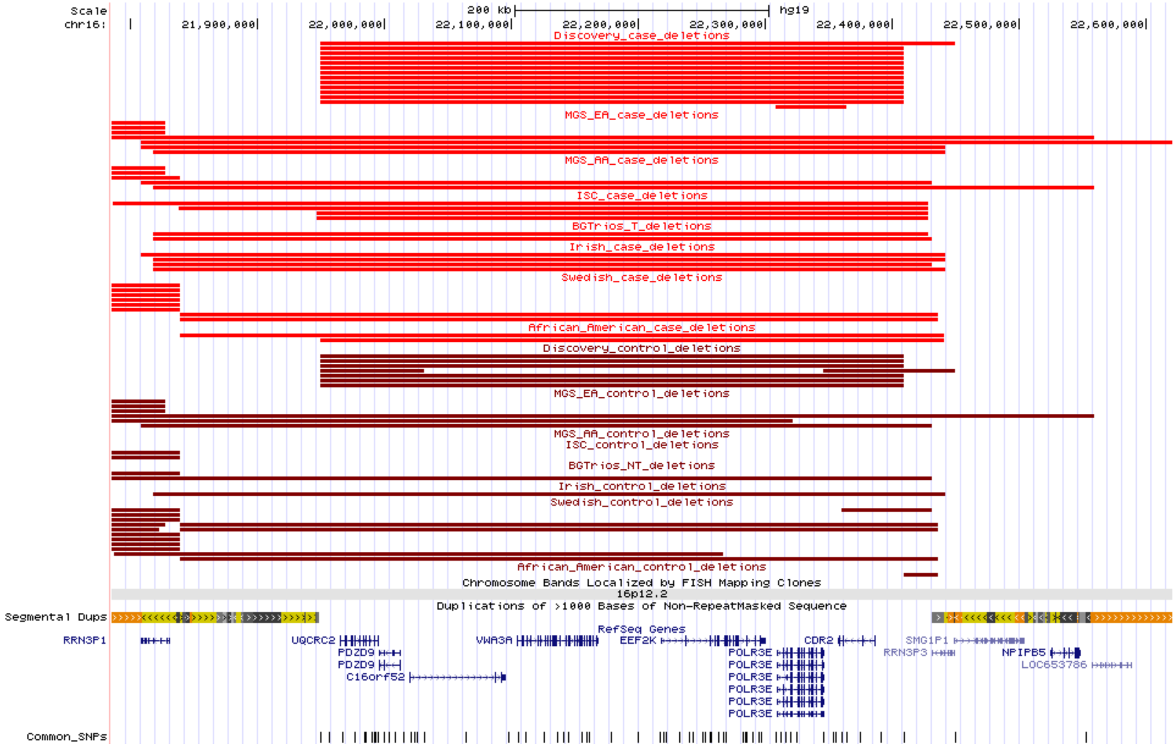

Figure S12. Deletions at 16p12.1

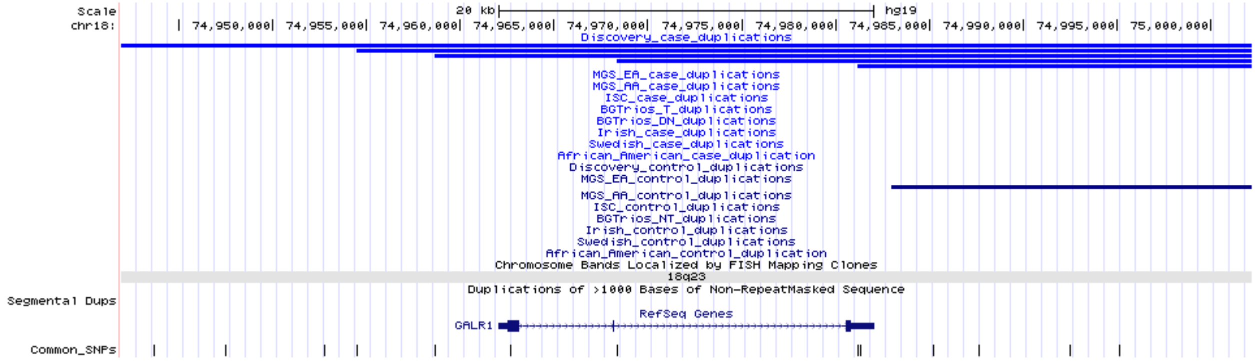

Figure S13. Duplications of *GALR1*

6. Acknowledgments

Members of Wellcome Trust Case Control Consortium 2

Management Committee

Peter Donnelly (Chair)[1,2], Ines Barroso (Deputy Chair)[3], Jenefer M Blackwell[4, 5], Elvira Bramon[6] , Matthew A Brown[7] , Juan P Casas[8] , Aiden Corvin[9], Panos Deloukas[3], Audrey Duncanson[10], Janusz Jankowski[11], Hugh S Markus[12], Christopher G Mathew[13], Colin NA Palmer[14], Robert Plomin[15], Anna Rautanen[1], Stephen J Sawcer[16], Richard C Trembath[13], Ananth C Viswanathan[17], Nicholas W Wood[18]

Data and Analysis Group

Chris C A Spencer[1], Gavin Band[1], Céline Bellenguez[1], Colin Freeman[1], Garrett Hellenthal[1], Eleni Giannoulatou[1], Matti Pirinen[1], Richard Pearson[1], Amy Strange[1], Zhan Su[1], Damjan Vukcevic[1], Peter Donnelly[1,2]

DNA, Genotyping, Data QC and Informatics Group

Cordelia Langford[3], Sarah E Hunt[3], Sarah Edkins[3], Rhian Gwilliam[3], Hannah Blackburn[3], Suzannah J Bumpstead[3], Serge Dronov[3], Matthew Gillman[3], Emma Gray[3], Naomi Hammond[3], Alagurevathi Jayakumar[3], Owen T McCann[3], Jennifer Liddle[3], Simon C Potter[3], Radhi Ravindrarajah[3], Michelle Ricketts[3], Matthew Waller[3], Paul Weston[3], Sara Widaa[3], Pamela Whittaker[3], Ines Barroso[3], Panos Deloukas[3].

Publications Committee

Christopher G Mathew (Chair)[13], Jenefer M Blackwell[4,5], Matthew A Brown[7], Aiden Corvin[9], Chris C A Spencer[1]

1 Wellcome Trust Centre for Human Genetics, University of Oxford, Roosevelt Drive, Oxford OX3 7BN, UK; 2 Dept Statistics, University of Oxford, Oxford OX1 3TG, UK; 3 Wellcome Trust Sanger Institute, Wellcome Trust Genome Campus, Hinxton, Cambridge CB10 1SA, UK; 4 Telethon Institute for Child Health Research, Centre for Child Health Research, University of Western Australia, 100 Roberts Road, Subiaco, Western Australia 6008; 5 Cambridge Institute for Medical Research, University of Cambridge School of Clinical Medicine, Cambridge CB2 0XY, UK; 6 Department of Psychosis Studies, NIHR Biomedical Research Centre for Mental Health at the Institute of Psychiatry, King's College London and The South London and Maudsley NHS Foundation Trust, Denmark Hill, London SE5 8AF, UK; 7 University of Queensland Diamantina Institute, Brisbane, Queensland, Australia; 8 Dept Epidemiology and Population Health, London School of Hygiene and Tropical Medicine, London WC1E 7HT and Dept Epidemiology and Public Health, University College London WC1E6BT, UK; 9 Neuropsychiatric Genetics Research Group, Institute of MolecularMedicine, Trinity College Dublin, Dublin 2, Eire; 10 Molecular and Physiological Sciences, The Wellcome Trust, London NW1 2BE; 11 Department of Oncology, Old Road Campus, University of Oxford, Oxford OX3 7DQ, UK , Digestive Diseases Centre, Leicester Royal Infirmary, Leicester LE7 7HH, UK and Centre for Digestive Diseases, Queen Mary University of London, London E1 2AD, UK; 12 Clinical Neurosciences, St George's University of London, London SW17 0RE; 13 King's College London Dept Medical and Molecular Genetics, King's Health Partners, Guy's Hospital, London SE1 9RT, UK; 14 Biomedical Research Centre, Ninewells Hospital and Medical School, Dundee DD1 9SY, UK; 15 King's College London Social, Genetic and Developmental Psychiatry Centre, Institute of Psychiatry, Denmark Hill, London SE5 8AF, UK; 16 University of Cambridge Dept Clinical Neurosciences, Addenbrooke's Hospital, Cambridge CB2 0QQ, UK; 17 NIHR Biomedical Research Centre for Ophthalmology, Moorfields Eye Hospital NHS Foundation Trust and UCL Institute of Ophthalmology, London EC1V 2PD, UK; 18 Dept Molecular Neuroscience, Institute of Neurology, Queen Square, London WC1N 3BG, UK.

7. References

1. Hamshere, M.L., Walters, J.T.R., Smith, R., Richards, A.L., Green, E., Grozeva, D., Jones, I., Forty, L., Jones, L., Gordon-Smith, K., et al. (2012) Genome-wide significant associations in schizophrenia to ITIH3/4, CACNA1C and SDCCAG8, and extensive replication of associations reported by the Schizophrenia PGC. *Mol Psychiatry*, **18**, 708-712.
2. Kirov, G., Rees, E., Walters, T.J., Escott-Price, V., Georgieva, L., Richards, A.L., Chambert, K.D., Davies, G., Legge, S.E., Moran, J.L., et al. (2013) The penetrance of copy number variations for schizophrenia and developmental delay. *Biol. Psychiatry.*, http://dx.doi.org/10.1016/j.biopsych.2013.1007.1022.
3. Rees, E., Walters, J.T.R., Georgieva, L., Isles, A.R., Chambert, K.D., Richards, A.L., Mahoney-Davies, G., Legge, S.E., Moran, J.L., McCarroll, S.A., et al. (2013) Analysis of copy number variations at 15 schizophrenia-associated loci. *Br J Psychiatry*, In press.
4. Wing, J.K., Babor, T., Brugha, T., Burke, J., Cooper, J.E., Giel, R., Jablenski, A., Regier, D., Sartorius, N. (1990) SCAN: Schedules for Clinical Assessment in Neuropsychiatry. *Arch. Gen. Psychiatry.*, **47**, 589-593.
5. American Psychiatric Association. Diagnostic and Statistical Manual of Mental Disorders. Fourth ed. Washington, DC.: American Psychiatric Press; 1994.
6. Levinson, D.F., Duan, J., Oh, S., Wang, K., Sanders, A.R., Shi, J., Zhang, N., Mowry, B.J., Olincy, A., Amin, F., et al. (2011) Copy number variants in schizophrenia: confirmation of five previous findings and new evidence for 3q29 microdeletions and VIPR2 duplications. *Am J Psychiatry*, **168**, 302-316.
7. Korn, J.M., Kuruvilla, F.G., McCarroll, S.A., Wysoker, A., Nemesh, J., Cawley, S., Hubbell, E., Veitch, J., Collins, P.J., Darvishi, K., et al. (2008) Integrated genotype calling and association analysis of SNPs, common copy number polymorphisms and rare CNVs. *Nat Genet*, **40**, 1253-1260.
8. International Schizophrenia Consortium (ISC). (2008) Rare chromosomal deletions and duplications increase risk of schizophrenia. *Nature*, **455**, 237-241.
9. Irish Schizophrenia Genomics Consortium and the Wellcome Trust Case Control Consortium 2. (2012) Genome-Wide Association Study Implicates HLA-C*01:02 as a Risk Factor at the Major Histocompatibility Complex Locus in Schizophrenia. *Biol. Psychiatry*, **72**, 620-628.
10. Ripke, S., O'Dushlaine, C., Chambert, K., Moran, J.L., Kähler, A., Akterin, S., Bergen, S., Collins, A.L., Crowley, J., Fromer, M., et al. (Submitted) Genome-wide association of schizophrenia in Sweden.
11. Kristjansson, E., Allebeck, P., Wistedt, B. (1987) Validity of the diagnosis of schizophrenia in a psychiatric inpatient register. *Nordisk Psykiatrik Tidsskrift*, **41**, 229-234.
12. Dalman, C., Broms, J., Cullberg, J., Allebeck, P. (2002) Young cases of schizophrenia identified in a national inpatient register--are the diagnoses valid? *Social Psychiatry and Psychiatric Epidemiology*, **37**, 527-531.
13. World Health Organization. International Classification of Diseases. 8th revised ed. Geneva: World Health Organization; 1967.
14. World Health Organization. International Classification of Diseases. 9th revised ed. Geneva: World Health Organization; 1978.
15. World Health Organization. International Classification of Diseases. 10th revised ed. Geneva: World Health Organization; 1992.
16. Hultman, C.M., Sparen, P., Takei, N., Murray, R.M., Cnattingius, S. (1999) Prenatal and perinatal risk factors for schizophrenia, affective psychosis, and reactive psychosis of early onset: case-control study. *Bmj*, **318**, 421-426.

17. Zammit, S., Allebeck, P., Dalman, C., Lundberg, I., Hemmingsson, T., Lewis, G. (2003) Investigating the association between cigarette smoking and schizophrenia in a cohort study. *Am J Psychiatry*, **160**, 2216-2221.
18. Andersson, R.E., Olaison, G., Tysk, C., Ekbom, A. (2001) Appendectomy and protection against ulcerative colitis. *N Engl J Med*, **344**, 808-814.
19. Hansson, L.E., Nyren, O., Hsing, A.W., Bergstrom, R., Josefsson, S., Chow, W.H., Fraumeni, J.F., Jr., Adami, H.O. (1996) The risk of stomach cancer in patients with gastric or duodenal ulcer disease. *N Engl J Med*, **335**, 242-249.
20. Schwartz, S., Susser, E. (2010) Genome-wide association studies: does only size matter? *Am J Psychiatry*, **167**, 741-744.
21. Craddock, N., Owen, M.J. (2010) The Kraepelinian dichotomy - going, going... but still not gone. *Br J Psychiatry*, **196**, 92-95.
22. International Schizophrenia Consortium. (2009) Common polygenic variation contributes to risk of schizophrenia and bipolar disorder. *Nature*, **460**, 748-752.
23. Lichtenstein, P., Yip, B., Bjork, C., Pawitan, Y., Cannon, T.D., Sullivan, P.F., Hultman, C.M. (2009) Common genetic influences for schizophrenia and bipolar disorder: A population-based study of 2 million nuclear families. *Lancet*, **373**, 234-239.
24. Purcell, S., Neale, B., Todd-Brown, K., Thomas, L., Ferreira, M.A.R., Bender, D., Maller, J., Sklar, P., de Bakker, P.I.W., Daly, M.J., et al. (2007) PLINK: A Tool Set for Whole-Genome Association and Population-Based Linkage Analyses. *Am J Hum Genet*, **81**, 559-575.
25. Kirov, G., Pocklington, A.J., Holmans, P., Ivanov, D., Ikeda, M., Ruderfer, D., Moran, J., Chambert, K., Toncheva, D., Georgieva, L., et al. (2012) De novo CNV analysis implicates specific abnormalities of postsynaptic signalling complexes in the pathogenesis of schizophrenia. *Mol. Psychiatry*, **17**, 142-153.
